# Development, Characterization, and Evaluation as Food Active Packaging of Low-Density-Polyethylene-Based Films Incorporated with Rich in Thymol Halloysite Nanohybrid for Fresh “Scaloppini” Type Pork Meat Fillets Preservation

**DOI:** 10.3390/polym15020282

**Published:** 2023-01-05

**Authors:** Aris E. Giannakas, Constantinos E. Salmas, Dimitrios Moschovas, Vassilios K. Karabagias, Ioannis K. Karabagias, Maria Baikousi, Stavros Georgopoulos, Areti Leontiou, Katerina Katerinopoulou, Nikolaos E. Zafeiropoulos, Apostolos Avgeropoulos

**Affiliations:** 1 Department of Food Science and Technology, University of Patras, 30100 Agrinio, Greece; 2 Department of Material Science and Engineering, University of Ioannina, 45110 Ioannina, Greece

**Keywords:** low-density polyethylene, halloysite nanotubes, thyme oil, active food packaging, controlled release, antioxidant activity, TBARS, heme iron, pork meat preservation, hybrid nanostructure

## Abstract

A new era is rising in food packaging and preservation, with a consequent focus on transition to “greener” and environmentally friendly techniques. The environmental problems that are emerging nowadays impose use of natural materials for food packaging applications, replacement of chemical preservatives with natural organic extractions, such as essential oils, and targeting of new achievements, such as further extension of food shelf-life. According to this new philosophy, most of the used materials for food packaging should be recyclable, natural or bio-based, and/or edible. The aim of this work was to investigate use and efficiency of a novel food packaging developed based on commercial LDPE polymer incorporated with natural material halloysite impregnated with natural extract of thyme oil. Moreover, a direct correlation between the stiff TBARS method and the easiest heme iron measurements method was scanned to test food lesions easier and faster. The result of this study was development of the LDPE/10TO@HNT film, which contains the optimum amount of a hybrid nanostructure and is capable to be used as an efficient active food packaging film. Furthermore, a linear correlation seems to connect the TBARS and heme iron measurements.

## 1. Introduction

The TBARS method is a commonly used and accurate procedure for estimation of food lesions grade. Heme iron is a physical index of blood that can be easily measured and could be directly correlated with TBARS level. Extending a previous work [1], which deals with development of a novel active packaging film using the biodegradable but not commercially used at this time polymeric matrix PVOH, in this work, we investigated if the same novel technique with the same novel nanohybrid additives could be combined with the most commercially used polymeric matrix, i.e., low-density polyethylene (LDPE), for active packaging film development. Moreover, we investigated the kind of correlation between TBARS and heme iron measurements. LDPE is one of the most used flexible food packaging films [2]. Due to its good moisture barrier properties and excellent thermomechanical properties, it can be used in thermal extrusion and co-extrusion industrial processes for manufacturing flexible films. LDPE biobased polymer, which originated from bio-polyethylene (bio-PE), is a dominant candidate for use in future industrial food packaging applications. Bio-PE is produced via a dehydration process of bioethanol to transform it into ethylene. Bioethanol is obtained via biochemical reactions, such as alcoholic fermentation, using various biomass feedstocks, including sugar cane, sugar beet, and wheat grain [3].

The current trend in food processing and safety leads to replacement of the old preservation process, i.e., direct addition of chemical preservatives on food, with a new one that enables addition of biodegradable natural preservatives to the packaging of the food to ensure their controlled release into the food [4,5]. One of the most promising natural additives that is used in active food packaging is essential oils (EOs) [6]. EOs attracted researchers’ interest because of their antioxidant, antimicrobial, and antifungal activity [7,8]. Thymol (TO) and other ingredients of thyme oil (*T. vulgaris*) include general recognized as safe (GRAS) material and a very common natural agent that was used often as a food additive for preservation [9]. According to the literature, different geographical origination places of plant extraction may influence *T. vulgaris* toxicity. In an acute toxicity assay in mice, the oil from Tlemcen exhibits a toxic effect at 4500 mg/kg doses and the oil from Mostaganem exhibits no toxicity even at 5000 mg/kg [10]. To overcome any toxicological fears, it is suggested to incorporate essential oils into the packaging and their controlled release in food, which significantly reduces the final amount released into the food [6]. However, such preservatives exhibit a drawback, which is their volatile nature and consequent easy EOs loss from the packaging material. This fact motivated researchers’ efforts to develop different encapsulation technologies of EOs in food packaging materials and stabilizing their controlled release in food [11,12,13,14]. Valderrama Solano and de Rojas Gante [12] developed antimicrobial packaging materials by incorporating known concentrations (*w*/*w*) of oregano and thyme oil into LDPE via ionizing treatment and directly by extrusion. Comparison tests of the two processes showed that, by extrusion process, they obtained films with 4 %wt. essential oils concentration that exhibited a higher inhibitory effect than the films obtained using the ionizing treatment. Recently, researchers proposed adsorption of EOs in nanoclays, such as montmorillonite (Mt) and Halloysite nanotubes (HNT), to develop a hybrid EO/nanoclay nanostructure and incorporation of such hybrids into the LDPE matrix [15,16,17,18,19]. Nanoclays such as HNT act as nanoreinforcements, barrier agents, and EOs nanocarriers according to this method. Mahdi et al. [17] performed impregnation under vacuum to load limonene into five different mineral nanocarriers, i.e., HNT, kaolinite, mesoporous silica nanoparticles, zinc oxide nanoparticles, and molecular sieve type 4A. The obtained limonene–nanocarrier hybrids were incorporated into LDPE and short- and long-term release studies indicated that both HNT and mesoporous silica nanoparticles exhibited long-time release profiles of limonene from LDPE films. Kepker et al. [19] developed LDPE/ethylene vinyl alcohol copolymer antimicrobial films containing carvacrol by co-extrusion and multiplication technique. The micro-layering process was followed to produce films with up to 65 alternating layers. Carvacrol was melt-compounded with LDPE or loaded into HNT in a pre-compounding step prior to film production. The results show that carvacrol was incorporated into plastic polymers constructed of tailored multiple layers without losing their antimicrobial capacity. In a previous study, montmorillonite (Mt) and organically modified montmorillonite (OrgMt) were incorporated with thyme, oregano, and basil EOs in various nominal contents. The obtained EO/Mt and EO/OrgMt hybrids were melt-extruded with LDPE to develop LDPE active packaging films. Such films exhibited controllable and long-life antioxidant activity. The nominal composition of EO/Mt and EO/OrgMt nanohybrids added to LDPE fixed to 3 %wt.

In this study, we used thyme essential oil and HNT to develop rich in thymol (TO) HNT hybrid nanostructures (TO@HNT). The incorporation process was based on a green distillation/evaporation method. The obtained TO@HNT nanohybrids were loaded in LDPE via a melt-extrusion preparation process at 5, 10, and 15 %wt. nominal contents, which were higher compared to those reported in the literature [15,16,17,18,19]. Such high contents of HNT and/or TO@HNT nanofillers, to the best of our knowledge, are reported for the first time. As reference material, LDPE/xHNT films were prepared without thymol. The obtained LDPE/xHNT and LDPE/xTO@HNT films were characterized morphologically with XRD analysis, FTIR spectroscopy, and SEM images. Thermogravimetric characterization was carried out by TG and DSC analysis, while tensile properties, water/oxygen barrier properties, and antioxidant activity of films were also studied. The main target of this study was to achieve in one LDPE/xTO@HNT film optimum thermal, mechanical, barrier, and antioxidant properties. Finally, this most active LDPE/xTO@HNT film was tested as active packaging film, which extends the self-life of “scaloppini” type fresh pork meat fillets. Active packaging indicators were used, with the lipid oxidation of the samples according to thiobarbituric-acid-reacting substances (TBARS) assay and heme iron content determination. Additional effort was exerted to determine any possible correlations between TBA and heme iron contents methods. This possible correlation will assist development of easier, costless, and rapid tests for lipid oxidation estimation of this type of pork meat based on measuring of the heme iron value and directly correlating it with TBA values. It will be the first time that a correlation between heme iron and TBA values will be reported for this type of pork meat fillets.

## 2. Materials and Methods

### 2.1. Materials

Sigma-Aldrich was the supplier of the halloysite nanotubes (Cas. No. 1332-58-7, Sigma-Aldrich, St. Louis, MO, USA) and LDPE (CAS No. 9002-88-4, Sigma-Aldrich, St. Louis, MO, USA) with Melt Index = 25 g/10 min (190 °C/2.16 kg) and density 0.915 g cm^−3^. Thyme oil was purchased from a local pharmacy market and produced by Chemco (Via Achille Grandi, 13-13/A, 42,030 Vezzano sul Crostolo RE, Italy). Fresh pork fillets “scaloppini type” was a kind offering of Aifantis Company (Aifantis Group–Head Quarters, Acheloos Bridge, Agrinio, Greece 30100). Three fresh and deboned pork fillets “scaloppini type” with approximate weight 700 g each were provided by a local meat processing plant Aifantis Company within one hour after slaughtering.

### 2.2. Preparation of Reach in Thymol TO@HNT Nanohybrids

For preparation of rich in thymol modified TO@HNT nanohybrid, a “green” distillation/evaporation method was applied. First, the HNT was vacuum-dried for 24 h. Then, 20 mL of thyme oil was placed in the spherical flask of a glass distillation apparatus and the distillation process took place at 190 °C and produced 7 mL fraction rich in D-limonene and L-cymene [20]. The flask with the remaining rich in thymol (TO) amount of 13 mL was placed in an electric heater, and on top of flask mouth a vertical glass tube included 2 g of vacuum dried HNT bed was mounted. Above this assembly, another vertical reflux condenser was placed. The remaining rich in TO thyme oil was heated at 300 °C and the rich in thymol molecules were evaporated and adsorbed in HNT. When the HNT bed turned color from white to brown, the process stopped and the produced modified HNT stored for further use.

### 2.3. Preparation of LDPE/xHNT and LDPE/xTO@HNT Films

For preparation of LDPE/xHNT and LDPE/xTO@HNT films, a minilab, twin, co-rotating extruder (Haake Mini Lab II, ThermoScientific, ANTISEL, S.A., Athens, Greece) was used. For melt extrusion processing, the uniform operating temperature was 140 °C at a screw speed of 100 rpm and for 5 min total time. HNT or TO@HNT material was added into LDPE pellets at final total contents of 5, 10, and 15 %wt. The obtained films were labeled as LDPE/5HNT, LDPE/10HNT, LDPE/15HNT, LDPE/5TO@HNT, LDPE/10TO@HNT, and LDPE/15TO@HNT. Pure LDPE pellets also extruded under the same processing conditions to obtain pure LDPE films.

### 2.4. XRD Analysis

XRD analysis of pure HNT and modified TO@HNT powders as well as all obtained LDPE/xHNT and LDPE/xTO@HNT films was performed with a Brüker D8 Advance diffractometer (Brüker, Analytical Instruments, S.A., Athens, Greece) with a LINXEYE XE high-resolution energy dispersive detector.

### 2.5. FTIR Spectroscopy

FTIR spectrometry was performed on pure thyme oil, pure HNT, and modified TO@HNT nanohybrid samples to investigate the chemical structure of the modified TO@HNT nanohybrid as well as the existence of possible interactions. FTIR analysis also performed in pure LDPE film as well as in LDPE/xHNT and LDPE/xTO@HNT films to investigate the interaction between the LDPE and HNT or TO@HNT materials. For the FTIR measurements, an FT/IR-6000 JASCO Fourier transform spectrometer (JASCO, Interlab, S.A., Athens, Greece) was used.

### 2.6. SEM Images

The surface morphology of pure LDPE film as well as LDPE/xHNT and LDPE/xTO@HNT films was investigated by carrying out SEM and EDX measurements using a JEOL JSM-6510 LV SEM Microscope (Ltd., Tokyo, Japan) equipped with an X-Act EDS-detector from Oxford Instruments, Abingdon, Oxfordshire, UK (an acceleration voltage of 20 kV was applied).

### 2.7. Thermogravimetric and Differential Analysis (TG-DTA)

Thermogravimetric (TGA and DTA) measurements were performed to all obtained LDPE/xHNT and LDPE/xTO@HNT films. The achieved TG-DTA plots of such materials were compared with those of pure LDPE films. A Perkin-Elmer Pyris Diamond TGA/DTA instrument (Interlab, S.A., Athens, Greece) was used. Samples of approximately 5 mg were heated under nitrogen atmosphere from 25 to 700 °C and with an increasing temperature rate of 5 °C/min.

### 2.8. Tensile Properties

Tensile measurements were carried out for all obtained LDPE/xHNT and LDPE/xTO@HNT films as well as for pure LDPE film according to the ASTM D638 method using a Simantzü AX-G 5kNt instrument (Simantzu. Asteriadis, S.A., Athens, Greece). Three to five samples of each film were tested at a crosshead speed of 2 mm/min. The samples were dumbbell-shaped with gauge dimensions of 10 mm × 3 mm × 0.22 mm. Force (N) and displacement (mm) were recorded during the test.

### 2.9. Water Barrier Properties

Water vapor transmission rate (WVTR) was measured for all LDPE/xHNT and LDPE/xTO@HNT films as well as for pure LDPE films according to ASTM E96/E 96M-05 method. To make our results comparable with others from different materials and with various film thickness, such WVTR values, which are water vapor mass transfer rate reduced per film cross-sectional area unit, were additionally processed to produce the D_WV_ values, which, according to Fick’s law, are the same rate but reduced further per unit of reverse film thickness and per unit of ΔC driving force opposite the film. The overall procedure for such measurements and calculations was reported in detail in previous publications [20,21].

### 2.10. Oxygen Barrier Properties

An oxygen permeation analyzer (O.P.A., 8001, Systech Illinois Instruments Co., Johnsburg, IL, USA) was used to measure the oxygen transmission rate (OTR) for all LDPE/xHNT and LDPE/xTO@HNT films as well as for pure LDPE film. To make our results comparable with others from different materials and with various film thickness, such OTR values, which are oxygen mass transfer rate reduced per film cross-sectional area unit and per unit of ΔC driving force opposite the film, were additionally processed to produce the Pe_O2_ values, which, according to gas permeability coefficient law, are the OTR but reduced further per unit of reverse film thickness. The overall procedure for such measurements and calculations was reported in detail in previous publications [20,21].

### 2.11. Determination of Fat Content of Scaloppini Pork Meat

The fatty substances were extracted using Soxhlet extraction. In particular, 15 ± 0.5 g of scaloppini fillets were placed in an extraction thimble of 30 mm × 100 mm size (Filtres Fioroni, Ingré, France) and introduced in the Soxhlet apparatus. The organic solvent used for the extraction was n-hexane (Merck, Darmstadt, Germany). The extraction process was completed in 4–6 h. The n-hexane was evaporated in an oven at 105 ± 1 °C and the fatty substances remained in the pre-weighed flask. The flask was weighed every 1–2 h until constant weight achieved (to ensure that there were no solvent residues remaining), cooled in a desiccator, and weighed again to calculate the value of fatty substances. The extraction process was carried out in three different samples (*n* = 3) and the mean value was expressed as g_fatty_/100 g_meat_ ± standard deviation.

### 2.12. Antioxidant Activity

For measurement of antioxidant activity of films, an amount of 500 mg of small pieces (approximately 3 mm × 3 mm) of each film was used. The overall procedure for such measurements and calculations was reported in detail in previous publications [20,21].

### 2.13. Packaging Preservation Test of “Scaloppini Type” Fresh Pork Meat Fillets

**Figure 1:**
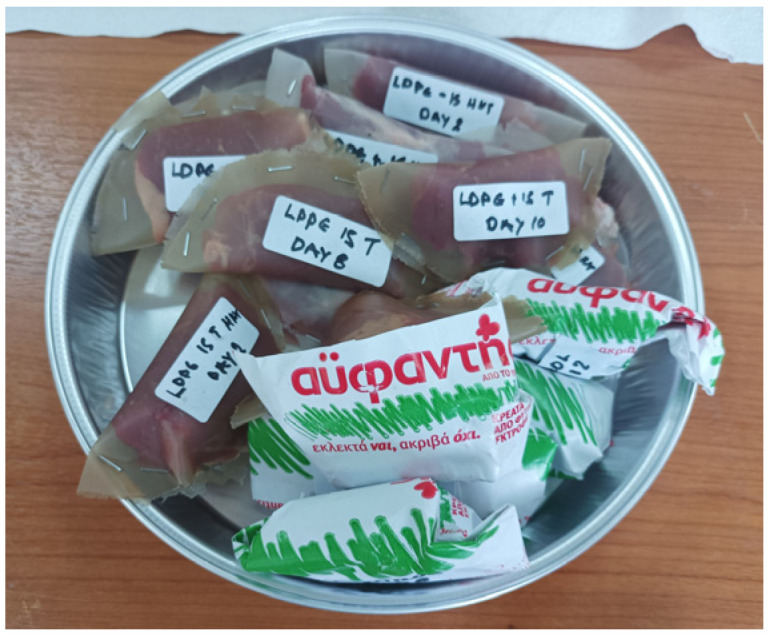
“Scaloppini” type fresh pork fillets after folded in LDPE or LDPE/xHNT or LDPE/xTO@HNT films and after packed with the official paper packaging of Aifantis Company.

Freshly slaughtered “scaloppini type” pork meat fillets were provided from a local meat processing plant “Aifantis” and transferred immediately in the lab. The pork fillets were aseptically cut in smaller pieces of 50 g each. Each piece of pork fillet was carefully folded into an LDPE/10TO@HNT active film with a diameter of 11 cm. Similar meat pieces were folded with LDPE/10HNT and pure LDPE films under the same conditions. Each folded pork fillet was put inside packaging paper of Aifantis Company, from which the inner polymeric membrane was removed (see Figure 1). Six different samples nominated for 2, 4, 6, 8, 10, and 12 days of preservation correspondingly were prepared for each tested film. After packaging, the fillets were placed in a preservation chamber at 5 ± 1 °C.

### 2.14. Lipid Oxidation of “Scaloppini” Type Fresh Pork Meat Fillets

#### 2.14.1. Thiobarbituric Acid Reactive Substances

ThioBarbituric acid reactive substances (TBARS) were determined according to [22] and Karabagias et al. [23]. Ten grams of “scaloppini” pork meat was macerated with 50 mL of deionized water for 2 min and transferred to a steam distillation flask with 47 mL of water and 2.5 mL of 4 M hydrochloric acid, which was added to adjust pH value to 1.5. After addition of a few glass beads, the flask was heated so as to collect 50 mL of distillate. Five mL of the distillate was pipetted into a glass-stoppered tube, 5 mL TBA reagent added, and the tube was stoppered, shaken, and heated in boiling baker for 35 min. A blank was prepared accordingly using 5 mL of water with 5 mL reagent. The tubes were cooled in water for 10 min and the absorbance was measured against the blank at λ = 538 nm (Abs_532_) using glass cuvettes of 1 cm.

TBARS was expressed as mg of malondialdehyde (mg_MDA_) in 1 kg of sample according to the following equation:

TBARS (mg_MDA_/kg_sample_) = 7.8 × Abs_538_ (1)

where 7.8 is a constant to transform the absorbance at λ = 538 nm to mg_MDA_/kg_sample_ [22].

#### 2.14.2. Heme Iron Content

The heme iron content was determined according to the method reported by Kalpalathika et al. [24] and [25]. In particular, 4 g of “scaloppini” pork meat was homogenized with a mixer (Vicko S.A, Athens, Greece) in 18 mL of acidified acetone. Then, the solution was left to stand at 25 °C, protected from light, for 1 h. Afterwards, the solution was filtered and absorbance was measured at 640 nm using a spectrophotometer. The amount of heme iron in the “scaloppini” pork meat was calculated according to the following equation:

Heme iron (μg_HFe_/g_sample_) = (Abs_640_ × 680) (μg_hematin_/g_sample_) × 10^−6^ (g_hematin_/μg_hematin_) × 88.2 (mg_HFe_/g_hematin_) × 10^3^ (μg_HFe_/mg_HFe_) OR HFe (μg_HFe_/g_sample_) = Abs_640_ × 680 × 0.0882 (2)

where Abs_640_ is the absorbance measured at 640 nm and 680 is a constant for conversion of absorbance at 640 nm to ppm concentration (μg_hematin_/g_sample_) if the prepared solution made following receipt of [24,25] and 0.0882 is the constant value to transform μg_hematin_ to μg_HFe_.

### 2.15. Statistical Analysis

Lipid oxidation results were subjected to one-way analysis of variance (ANOVA) with Bonferroni’s post-hoc test to indicate any statistical differences among the three packaging systems (control, HNT, and TO@HNT) on the TBARS and heme iron content values with respect to storage time. The least significance difference (LSD) was that of *p* < 0.05. The correlation of TBARS and heme iron method was estimated using Pearson’s bivariate correlation (−1 to +1) at the confidence level *p* < 0.05. Statistical analysis was completed using SPSS software (v. 28.0, IBM, Armonk, NY, USA).

## 3. Results

### 3.1. Characterization of Modified TO@HNT Nanohybrids

In a recent publication [1], XRD analysis, FTIR spectroscopy, TG analysis, and DSC measurements of pure HNT and modified TO@HNT nanohybrid showed that physisorption of a mixture rich in TO molecules took place on the external surface of HNT. The controlled release process of physiosorbed molecules is easier compared to that of chemisorbed molecules in such EOs–nanoclays hybrids. The average TO content of HNT was calculated equal to 34.5 %wt. Considering that the modified TO@HNT contains 34.5 %wt. TO, the calculated nominal content of TO in the obtained LDPE/5TO@HNT, LDPE/10TO@HNT, and LDPE/15TO@HNT active films is 1.725 %wt., 3.45 %wt., and 5.175 %wt., respectively.

### 3.2. XRD Analysis of LDPE/xHNT and LDPE/xTO@HNT Films

**Figure 2:**
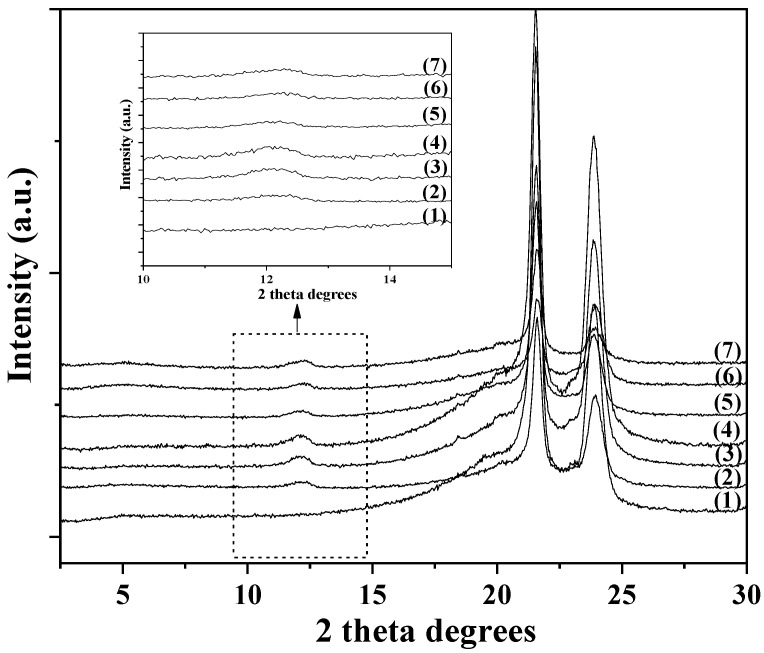
XRD plots in the range of 2 theta 2.5° to 30° of (1) pure LDPE, (2) LDPE/5HNT, (3) LDPE/10HNT, (4) LDPE/15HNT, (5) LDPE/5TO@HNT, (6) LDPE/10TO@HNT, and (7) LDPE/15TO@HNT. Inset figure with the same XRD plots in the range of 2 theta 10° to 15°.

The XRD plots of all LDPE/xHNT and LDPE/xTO@HNT films as well as the XRD plot of pure LDPE film are shown in Figure 2.

Pure LDPE film has two crystallization peaks at 2 theta 21.6° and 23.8°. Increasing HNT or TO@HNT %wt. content in LDPE/xHNT or LDPE/xTO@HNT films, the LDPE’s characteristic peaks decrease. Moreover, the characteristic peak of HNT basal space for all LDPE/xHNT and LDPE/xTO@HNT films is obtained at 12.1°. This implies that no opening of HNT’s basal space is achieved, and HNT or TO@HNT nanostructures are uniformly dispersed inside LDPE’s chains. Moreover, in the inset graph of Figure 2, the XRD plots exhibit a basal space peak at 12.1° for the HNTs, which is also observable for all LDPE/xHNT and LDPE/xTO@HNT films for higher analysis. For LDPE/xHNT films, it is observed that, as the %wt. HNT content increases, the peak slightly increases. This implies that, by increasing the %wt. HNT content, the aggregation of HNT increases and the dispersion of HNT decreases. In addition, the same peak of LDPE/xTO@HNT films is lower than that of LDPE/xHNT films. This implies that the dispersion of the modified TO@HNT in LDPE matrix is higher compared to the dispersion of the pure HNT in LDPE.

### 3.3. FTIR Spectroscopy of LDPE/xHNT and LDPE/xTO@HNT Films

**Figure 3:**
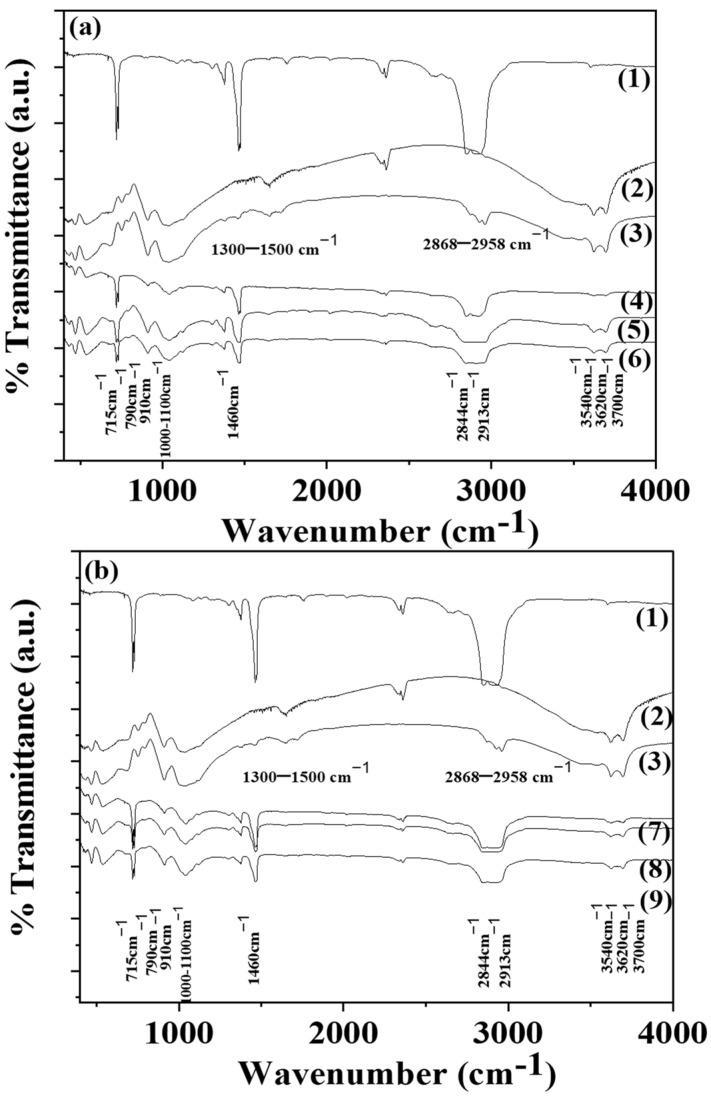
(**a**) FTIR plots of (1) pure LDPE, (2) pure HNT, (3) TO@HNT hybrid nanostructure, (4) LDPE/5HNT, (5) LDPE/10HNT, and (6) LDPE/15HNT films, (**b**) FTIR plots of (1) pure LDPE, (2) pure HNT, (3) TO@HNT hybrid nanostructure, (7) LDPE/5TO@HNT, (8) LDPE/10TO@HNT, and (9) LDPE/15TO@HNT.

The FTIR plots of pure LDPE (line (1)) of all LDPE/xHNT (see lines (4), (5), (6)) and all LDPE/xTO@HNT (see lines (7), (8), (9)) films are depicted in Figure 3. Moreover, the FTIR plots of pure HNT (line (2)) and TO@HNT hybrid nanostructure (line (3)) are included.

In the FTIR plot of pure LDPE (see line (1)), –CH_3_ asymmetric stretching, –CH_2_ wagging, and –CH_2_ rocking are depicted by peaks at 1460 and 715 cm^−1^. In addition,–CH_2_ symmetric stretching peaks are also observed at 2913 and 2844 cm^−1^ [26,27,28,29]. In the FTIR plot of both pure HNT and modified TO@HNT hybrid nanostructure (see lines (2) and (3)) are observed bands at 3700 and 3620 cm^−1^ of hydroxyl groups in the internal HNT’s surface. The weak band at 3540 cm^−1^ is of the Si–O–Si (Al) groups. The intense absorption bands in the region of 1100–1000 cm^−1^ and at 790 cm^−1^ are of Si–O group. The band at 910 cm^−1^ represents the hydroxyl group’s bending vibration. The band at 745 cm ^−1^ represents the Si–O–Al bonds [30,31]. In addition, on the FTIR plot of hybrid TO@HNT, some of the characteristic bands of thyme oil are also obtained [1,32]. These are the absorption bands in the range of 2958 to 2868 cm^−1^, which are assigned to the stretching mode of C-H groups, and the bands between 1500 cm^−1^ and 1300 cm^−1^, which are assigned to the C-H bending of the aliphatic CH_2_ groups and C-O-H groups bending [32]. In the FTIR plots of all LDPE/xHNT (see lines (4), (5), and (6)) and all LDPE/xTO@HNT (see lines (7), (8), and (9)) films, mixing of the characteristic plot of the LDPE (see line (1)) film with some of the characteristic peaks of the HNT material is obvious, i.e., at 790, 910, 1000–1100, 3620, and 3600 cm^−1^. Moreover, no shift in the LDPE’s characteristic peak is observed, which indicates good dispersion of both HNT and TO@HNT nanohybrids in the LDPE matrix [28,29]. It must be noticed that no characteristic peak of thyme oil is observed in all LDPE/xHNT and LDPE/xTO@HNT films, which implies that TO was adsorbed in the internal surface of the obtained LDPE/xTO@HNT films [1,20].

### 3.4. SEM

**Figure 4:**
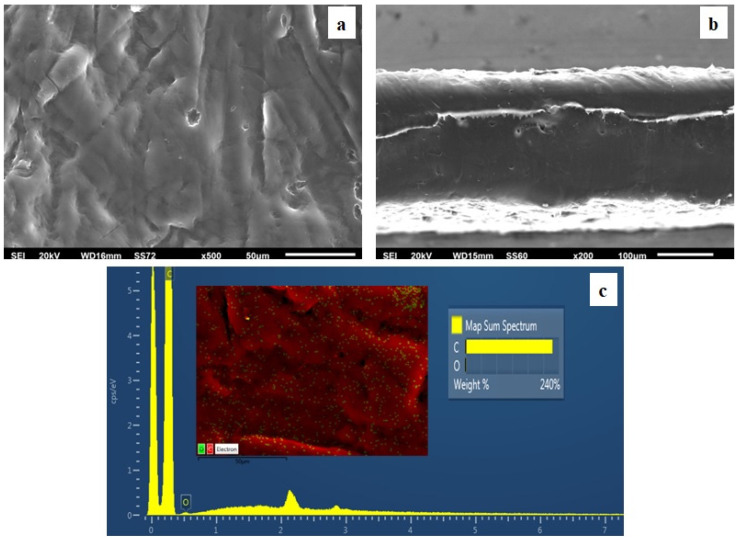
(**a**) SEM images of surface and (**b**) cross-section for the pure film of LDPE. (**c**) Energy dispersive spectrometer (EDS) spectra, relative elemental analysis, and mapping of the surface from the SEM image at ×1000 magnification.

**Figure 5:**
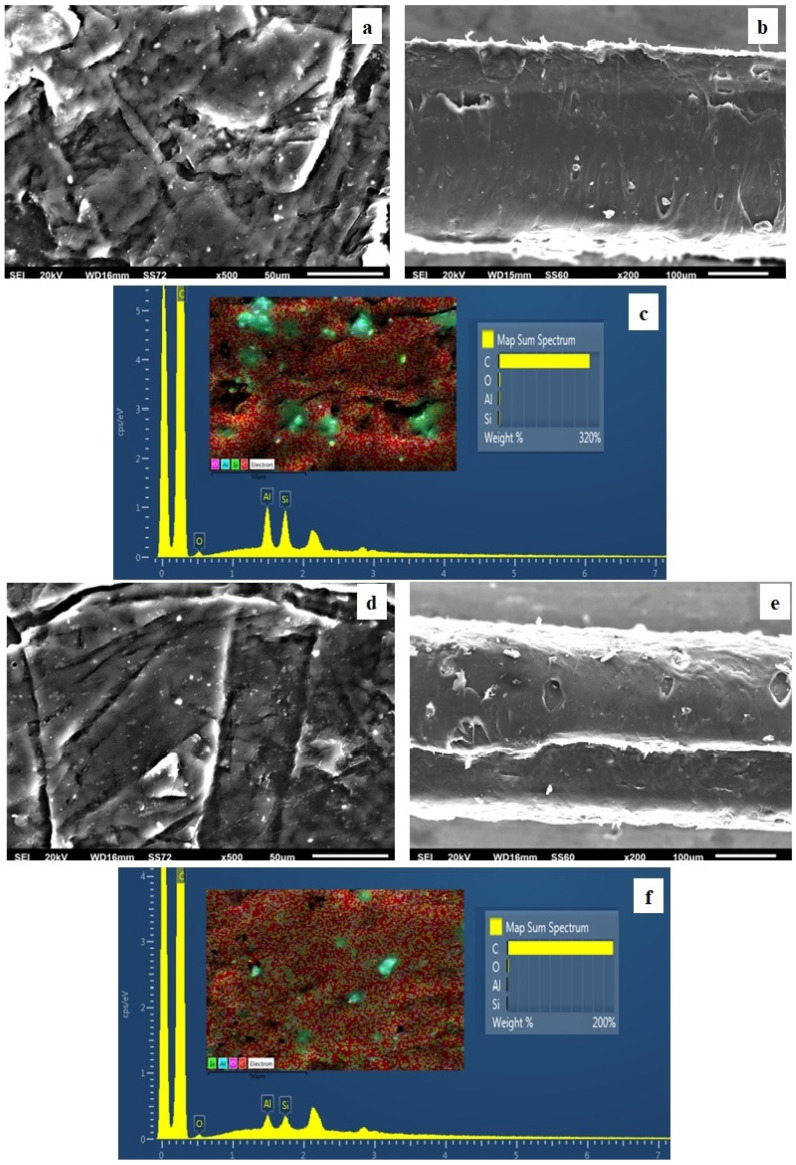
(**a**,**d**) SEM images of surface and (**b**,**e**) cross-section for the nanocomposite films of LDPE/5HNT (**a**,**b**) and LDPE/5TO@HNT (**d**,**e**), respectively. (**c**,**f**) Energy dispersive spectrometer (EDS) spectra, relative elemental analysis, and mapping of the surface from the SEM images at ×1000 magnification.

**Figure 6:**
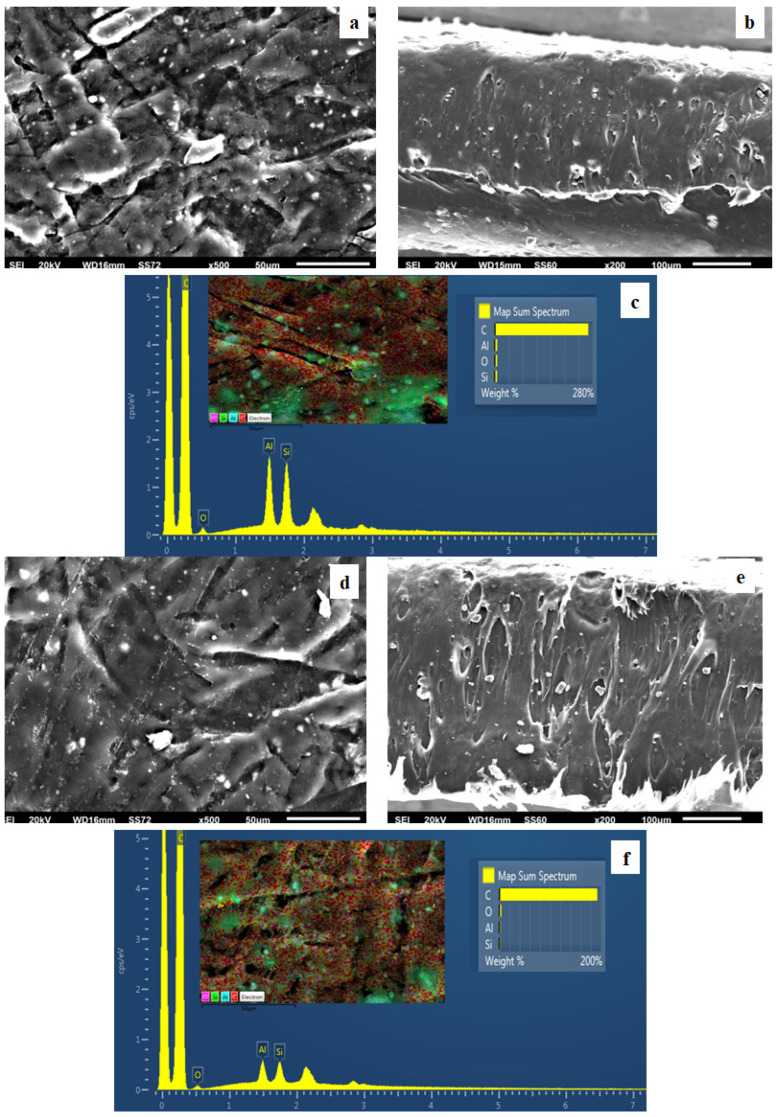
(**a**,**d**) SEM images of surface and (**b**,**e**) cross-section for the nanocomposite films of LDPE/10HNT (**a**,**b**) and LDPE/10TO@HNT (**d**,**e**), respectively. (**c**,**f**) Energy dispersive spectrometer (EDS) spectra, relative elemental analysis, and mapping of the surface from the SEM images at ×1000 magnification.

**Figure 7:**
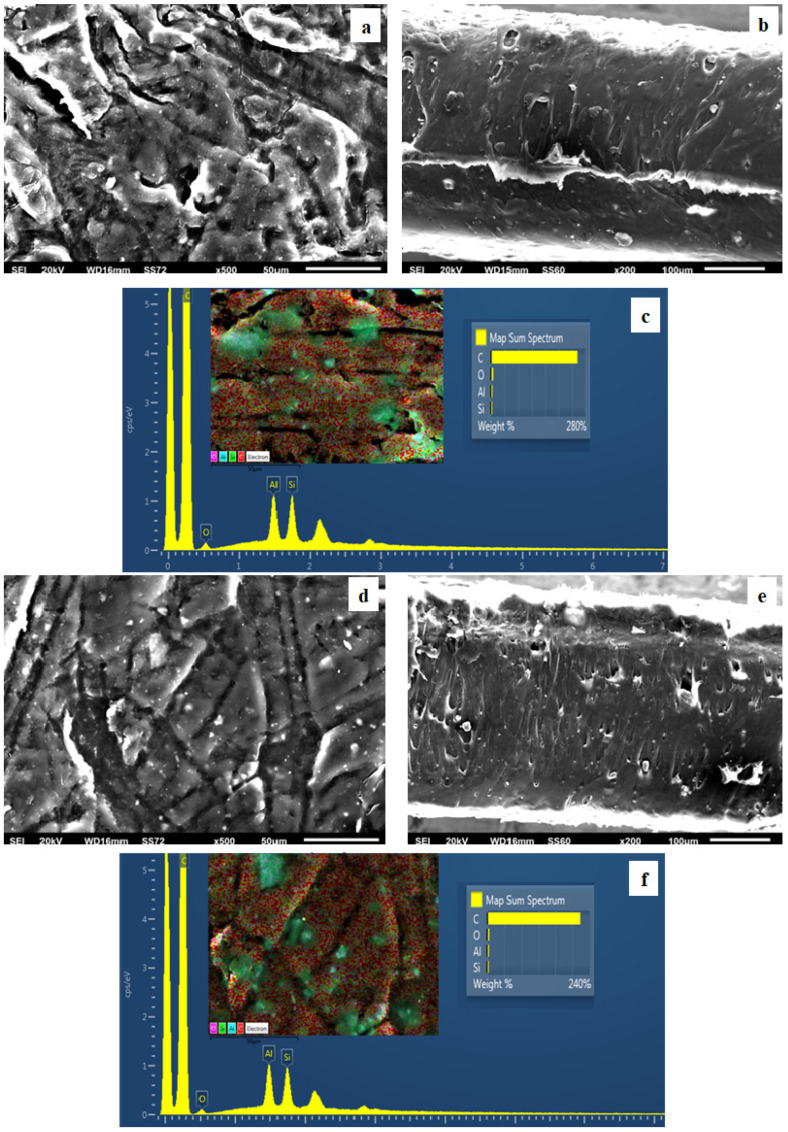
(**a**,**d**) SEM images of surface and (**b**,**e**) cross-section for the nanocomposite films of LDPE/15HNT (**a**,**b**) and LDPE/15TO@HNT (**d**,**e**), respectively. (**c**,**f**) Energy dispersive spectrometer (EDS) spectra, relative elemental analysis, and mapping of the surface from the SEM images at ×1000 magnification.

The surface/cross-section morphology of the pure polymer matrix LDPE and hybrid nanocomposite films LDPE/xHNT and LDPE/xTO@HNT was investigated using a SEM instrument equipped with an EDS detector. The results for all investigated films are shown in Figure 4, Figure 5, Figure 6 and Figure 7 for pure LDPE, LDPE/5HNT-LDPE/5TO@HNT, LDPE/10HNT-LDPE/10TO@HNT, and LDPE/15HNT-LDPE/15TO@HNT films, correspondingly.

The results confirmed that the HNT and hybrid nanostructure TO@HNT were homogeneously dispersed in the polymer matrix. EDS elemental analysis and mapping from the surface of the pure and nanocomposite active packaging films were recorded in order to identify the chemical elements of the final materials.

The SEM images in Figure 4a,b exhibit the expected smooth and clear morphology of the pristine polymer, and the EDS spectra in Figure 4c certify the existence of carbon © and oxygen (O).

In order to perform the contrast with nanocomposite active films, the EDS spectra in Figure 5c,f, Figure 6c,f, Figure 7c,f show a chemical analysis, which identified typical elements, such as Si, Al, and O, present in the packaging films with different concentrations (5, 10, 15%) of TO@HNT hybrid nanostructure and pure HNT (white dots can be clearly identified on the surface of LDPE).

Surface, relative cross-section, and chemical mapping images of LDPE/xHNT and LDPE/xTO@HNT films with different ratios of HNT and TO@HNT are presented in Figure 5, Figure 6 and Figure 7. It is obvious that an increase in contents (after incorporation of HNT and TO@HNT) in the nanocomposite materials increases the aggregation degree accordingly. Nevertheless, the results of the SEM studies of the final nanocomposite films confirmed that the nanohybrids were homogeneously dispersed, which indicates their enhanced compatibility with the polymer matrix.

It should be mentioned, based on the SEM studies (surface and cross-section) and chemical mapping, that significant differences are observed when TO@HNT hybrid nanostructure is incorporated in the polymer matrix. This happens because better interfacial adhesion and homogenous dispersion are taking place compared with the respective nanocomposite film with pure HNT.

### 3.5. TG Experiments

**Figure 8:**
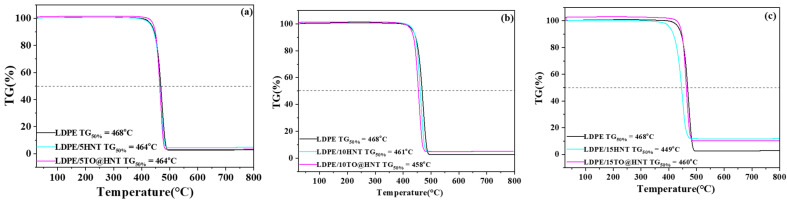
TG plots of (**a**) LDPE/5HNT, LDPE/5TO@HNT films, (**b**) LDPE/10HNT, LDPE/10TO@HNT films, and (**c**) LDPE/15HNT, LDPE/15TO@HNT films. In each graph, the TG plot of pure LDPE film is also presented for comparison.

TG plots of all LDPE/xHNT and LDPE/xTO@HNT films as well as of pure LDPE films are shown in Figure 8.

As is shown in the TG plots of Figure 8, all LDPE/xHNT and LDPE/xTO@HNT films as well as pure LDPE film exhibit one main degradation step, which starts at approximately 400 °C and ends at approximately 500 °C [28,29]. TG50% values are also provided by this figure. Such values correspond to the temperature at which 50% of mass was lost and are also indicative of the thermal strength of such films. It is obvious from these TG50% values that any addition of both pure HNT or TO@HNT hybrid nanostructure causes a slight decrease to the thermal strength of the obtained LDPE films. It is also obvious that, as the nanohybrid content increases, the residue at the end of the TG lines increases. This residue represents the HNT content. As an overall conclusion, considering the thermal stability, we can say that the LDPE/xHNT as well as the LDPE/xTO@HNT films do not exhibit serious disadvantages compared to pure LDPE.

### 3.6. Tensile Properties

In Table 1, the calculated values of Young’s (E) modulus, ultimate tensile strength (σ_uts_), and % strain at break (ε_b_) for all LDPE/xHNT and LDPE/xTO@HNT films as well as pure LDPE are listed.

It is obvious in Table 1 that, as the content of HNT increases, the values of elastic modulus (E) increase, while the strength (σ_uts_) and elongation at break (ε%) values decrease. This is typical behavior when rigid particles are added to the LDPE matrix [27,28]. On the contrary, when the TO@HNT hybrid nanostructure is added into the LDPE matrix, the values of both elastic modulus (E) and elongation at break (ε%) increase. For the LDPE/5TO@HNT and LDPE/15TO@HNT films, the strength values are lower in comparison to pure LDPE film, while, for the LDPE/10TO@HNT film, the strength value increases in comparison to pure LDPE film. Thus, the LDPE/10TO@HNT film has approximately 53%, 2.4%, and 95% higher stress, strength, and elongation at break values than the pure LDPE film. According to this result, we could say that the TO@HNT hybrid nanostructure achieves higher dispersions in LDPE matrix than the pure HNT. This result is depicted in the obtained tensile properties of such LDPE/xTO@HNT films. The presence of TO on TO@HNT nanohybrids acts as a plasticizer and improves the obtained elongation at break values of LDPE/xTO@HNT films. The higher dispersion of TO@HNT in the LDPE matrix compared to HNT is also obvious from the tensile properties of LDPE/xTO@HNT films. This observation is in accordance with SEM images analysis. Overall, the optimum stress, strength, and elongation at break values are achieved for the LDPE/10TO@HNT film.

### 3.7. Water Vapor Transmission Rate (WVTR) and Oxygen Transmission Rate (OTR)

In Table 2, the obtained values of WVTR and OTR of all LDPE/xHNT and LDPE/xTO@HNT films as well as of pure LDPE film are listed. From these values, the water vapor diffusion coefficient (D_wv_) and oxygen permeability (Pe_O2_) were calculated according to the procedure described in the literature [20,21] and listed in Table 2.

According to Table 2 data, comparing the D_wv_ and Pe_O2_ values of the LDPE/xHNT films (x = 5, 10, 15 %wt.) with those of pure LDPE film, addition of HNT initially causes an increase to the D_wv_ and Pe_O2_ values (i.e., for 5 %wt.) and sequentially causes a decrease to the D_wv_ and Pe_O2_ values (i.e., for 10 and 15 %wt.). When the TO@HNT hybrid nanostructure is added in LDPE, the obtained D_wv_ and Pe_O2_ values are lower than the Dwv and Pe_O2_ values of the pure LDPE. Moreover, such values of the LDPE/xTO@HNT films are lower than the relevant values of the LDPE/xHNT films. It seems that the TO@HNT nanostructure that originated from modification of the HNT with TO exhibits hydrophobic behavior and higher dispersion in the LDPE matrix. Moreover, according to the water vapor coefficient D_WV_ and the oxygen permeability coefficient Pe_O2_, both the water and oxygen barrier increased with modification of the HNT with TO. As an overall conclusion and according to the D_WV_ and Pe_O2_ values, the LDPE/5TO@HNT film exhibits a higher water vapor barrier, while the LDPE/10TO@HNT film exhibits a higher oxygen barrier. The lowest water/oxygen barrier is achieved from the LDPE/5HNT film.

### 3.8. Fat Content

The measurements carried out according to the procedure described in paragraph 2.11 resulted in the fat content of scaloppini pork meat fillets being 12.11 ± 1.52 g/100 g, indicating that it is a low-fat type of pork meat compared to the fat content of pork portions (fresh, whole, loin, etc.) (USDA Food Data Central, 2022) [33].

### 3.9. Antioxidant Activity

**Figure 9:**
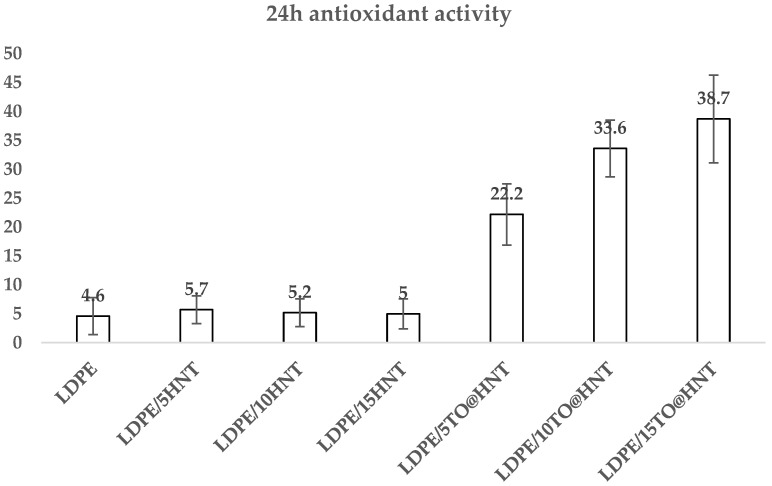
Antioxidant activity values after 24 h incubation for all LDPE/xHNT, LDPE/xTO@HNT films, as well as pure LDPE film.

In the column bar diagram of Figure 9, the calculated antioxidant activity values of all LDPE/xHNT, LDPE/xTO@HNT film, as well as pure LDPE film are presented.

It is obvious that the antioxidant activity of pure LDPE film and all LDPE/xHNT films is almost zero. In the case of LDPE/xTO@HNT films, the obtained antioxidant activity values increase as the %wt. content of TO@HNT nanohybrid increases.

### 3.10. Lipid Oxidation

#### 3.10.1. TBARS

The TBARS values for “scaloppini” pork meat samples packaged in control, HNT, and TO@HNT are shown in Table 3. The higher the value of TBARS, the higher the degree of deterioration in “scaloppini” pork meat. Such deterioration is a limiting factor for the shelf life of this type of meat or any other meat product. Karabagias et al. [23] reported that TBARS values of lamb meat packaged under modified atmosphere packaging did not exceed the value of 2 mg MDA/kg for samples during the first 10 days of storage. Zhang et al. [34] reported that perception of rancid taste due to lipid oxidation is recognized by consumers when the TBARS value is higher than the limit of 2.0 mg MDA/kg in beef.

As observed in Table 3, none of the treatments exceeded this limit highlighted in the literature during the 12 days of refrigerated storage. However, given the off odor that was developed, especially, during the 10th day of refrigerated storage, we propose the level of 1.2 mg MDA/kg in “scaloppini” pork meat as an index of lipid oxidation. In addition, the control treatment showed significantly (*p* < 0.05) higher TBARS values throughout the storage period. The delay in lipid oxidation was assessed by TO@HNT packaging followed by HNT, which had significantly (*p* < 0.05) lower values than the control samples. HNT reinforced with thyme had lower TBARS values, indicating antioxidant activity of thyme essential oil given its composition [23].

Active packaging has a beneficial effect on delay of lipid oxidation. In agreement with the results of the present study, Moudache et al. [35] reported that, in pork meat wrapped in films containing olive extract, there was recorded a delay on lipid oxidation. In a more recent study, Boeira et al. [36] also reported a significant reduction in the lipid oxidation of rump steak packaged in films containing corn stigma residue extracts.

#### 3.10.2. Heme Iron Content

The changes in heme iron content of “scaloppini” meat samples are shown in Table 2. The initial value (Day 0) of heme iron content for the “scaloppini” pork meat samples studied in the present study was 9.38 ± 0.09 μg/g. Previous studies [37,38] have reported that the heme iron content in different portions of pork meat (loin, topside, shoulder, and rump) ranged from 1.70 to 8.20 μg/g, in general agreement with the results of the present study.

Significant differences were observed (*p* < 0.05) between the control, HNT, and TO@HNT packaging films. The best results were obtained for the “scaloppini” pork meat samples packaged under TO@HNT in which a significantly (*p* < 0.05) smaller decrease in heme iron content was observed throughout storage time. Low values of heme iron, especially in the control samples, occurred due to release of iron caused by rupture of heme with an increase in refrigerated storage time. Lee et al. [39] reported that, during refrigerated storage of meat, there is a release of iron from the heme group, with a consequent increase in non-heme iron, which accelerates lipid oxidation. In the present study, only the control treatment had higher lipid oxidation, followed by HNT packaging treatment. The heme iron content of treatments HNT and TO@HNT was significantly (*p* < 0.05) higher when compared to the control throughout the refrigerated storage. These results indicate that the prepared films containing HNT and TO@HNT are effective against heme iron oxidation of “scaloppini” pork meat. Therefore, a limit value of 1.5 μg/g is proposed in the heme iron content as an index of lipid oxidation in “scaloppini” pork meat during refrigerated storage for 10 days. In a previous study [40], a delay of lipid oxidation was reported for beef samples packaged in protein films containing Lawsonia inermis extract.

#### 3.10.3. Correlation of TBARS and Heme Iron

The TBARS and heme iron content values throughout the storage time of “scaloppini” pork meat were subjected to Pearson’s correlation bivariate analysis. The results showed that a significant and negative correlation between the two methods was recorded for the whole storage time. The respective correlations were −0.929 (*p* < 0.001) for day 0, −0.927 (*p* < 0.001) for day 2, −0.934 (*p* < 0.001) for day 4, −0.969 (*p* < 0.001) for day 6, −0.974 (*p* < 0.001) for day 8, −0.946 (*p* < 0.001) for day 10, and −0.940 (*p* < 0.001) for day 12. Therefore, when the heme iron content linearly decreases, the TBARS values linearly increase. In this context, an analyst has the option to select which method to apply for estimation of lipid oxidation to gain time and reduce the cost of analyses.

## 4. Conclusions

In this study, we prepared an EOs solution enriched in thyme oil (TO) via a distillation process. Sequentially, a vapor-adsorption impregnation technique was followed to develop TO@HNT hybrid nanostructures. LDPE/xHNT and LDPE/xTO@HNT packaging films were prepared and tested for their mechanical and thermal properties, their water vapor and oxygen barrier, and their antioxidant and antimicrobial activity. Tensile tests indicated that, even though addition of HNT or TO@HNT causes a slight decrease to ultimate strength and elongation at break, there is a major enhancement to Young’s modulus, which means enhancement regarding mechanical properties. According to XRD and SEM-EDS results, the TO were adsorbed mainly on the external surface of the HNT and the nanostructures were dispersed uniformly in the LDPE polymeric matrix. Moreover, the SEM measurements indicated that there is a limit for the HNT or TO@HNT amount incorporated with the LDPE matrix, and, beyond this, aggregation somewhat begins. The FTIR measurements confirmed the physisorption mechanism of the HNT or TO@HNT on the LDPE surface, which make controlled release easier. The TG_50%_ indicator showed that any addition of HNT or TO@HNT decreases the thermal strength of films and there is a limit to allowed addition. The WVTR and OTR tests confirmed that TO increases the water vapor and oxygen barrier values as compared to the relevant ones with only HNT addition. A higher water vapor barrier was exhibited by the LDPE/5TO@HNT sample, while a higher oxygen barrier was exhibited by the LDPE/10TO@HNT sample. Samples with TO exhibited better water vapor or oxygen barriers as compared with samples containing only HNT. This happened probably because of the hydrophobic nature of the TO. Finally, the higher the content of TO, the higher the antioxidant activity, while sample LDPE/10TO@HNT contains the optimum amount of the hybrid nanostructure, as indicated from TBARS and heme iron measurements. Such measurements seem to be strongly correlated with a linear relationship. In conclusion, as an overall result, we could say that the LDPE/10TO@HNT film is a promising active packaging material, and the proposed process is ready for a scale-up step.

## Figures and Tables

**Table 1:** Calculated values of Young’s (E) modulus, ultimate tensile strength (σ_uts_), and % strain at break (ε_b_).

Sample Name	E	σ_uts_	ε%
LDPE	183.3 ± 4.8	12.6 ± 0.5	29.3 ± 1.1
LDPE/5HNT	210.0 ± 3.9	11.9 ± 0.5	15.0 ± 1.5
LDPE/10HNT	266.7 ± 1.3	12.0 ± 1.1	11.2 ± 0.8
LDPE/15HNT	301.7 ± 2.7	10.5 ± 3.4	16.9 ± 1.0
LDPE/5TO@HNT	298.3 ± 8.4	12.1 ± 1.3	39.5 ± 1.2
LDPE/10TO@HNT	280.3 ± 3.8	12.9 ± 1.4	57.1 ± 2.8
LDPE/15TO@HNT	250.3 ± 4.6	11.2 ± 0.9	21.4 ± 1.5

**Table 2:** Film thickness, WVTR, D_wv_, OTR, and Pe_O2_ values of all LDPE/xHNT and LDPE/xTO@HNT films as well as pure LDPE film.

Sample Name	Film Thickness (mm)	Water Vapor Transmission Rate (10^−7^ g × cm^−2^ × day^−1^)	Water Vapor Diffusion Coefficient D_wv_ (10^−4^ cm^2^ × s^−1^)	Oxygen Transmission Rate (mL × m^−2^ × day^−1^)	Oxygen Permeability Pe_O2_ (10^−8^ cm^2^ × s^−1^)
LDPE	0.241 ± 0.004	3.67 ± 0.74	3.05 ± 0.40	6407 ± 320	17.9 ± 0.84
LDPE/5HNT	0.413 ± 0.015	6.90 ± 0.64	6.57 ± 0.56	3866.0 ± 413	18.4 ± 0.92
LDPE/10HNT	0.306 ± 0.011	4.70 ± 0.14	3.29 ± 0.84	2822 ± 141	9.9 ± 0.45
LDPE/15HNT	0.313 ± 0.013	3.95 ± 1.73	2.85 ± 0.12	3035 ± 151.0	11.1 ± 0.55
LDPE/5TO@HNT	0.160 ± 0.038	3.29 ± 0.42	1.20 ± 0.23	8670 ± 433.5	16.1 ± 0.81
LDPE/10TO@HNT	0.281 ± 0.010	3.00 ± 0.80	1.94 ± 0.48	938.7 ± 47	3.1 ± 0.15
LDPE/15TO@HNT	0.207 ± 0.012	5.69 ± 0.22	2.67 ± 0.91	3616 ± 180	8.7 ± 0.43

**Table 3:** TBARS and heme iron content of “scaloppini” pork meat in different packaging systems with respect to storage time.

TBARS	Day 0	Day 2	Day 4	Day 6	Day 8	Day 10	Day 12
	AVG ± SD						
	(mg/kg)						
Control	0.20 ± 0.01	0.29 ± 0.01	0.50 ± 0.01	0.75 ± 0.02	1.03 ± 0.02	1.21 ± 0.02	1.34 ± 0.02
LDPE/10HNT	-	0.25 ± 0.01	0.40 ± 0.00	0.60 ± 0.01	0.89 ± 0.03	1.08 ± 0.01	1.27 ± 0.01
LDPE/10TO@HNT	-	0.22 ± 0.00	0.36 ± 0.01	0.49 ± 0.01	0.80 ± 0.01	1.00 ± 0.00	1.20 ± 0.03
	ANOVA						
F	-	45.750	141.867	264.704	82.616	301.412	31.826
*p*	-	0.000	0.000	0.000	0.000	0.000	0.001
Fe	Day 0	Day 2	Day 4	Day 6	Day 8	Day 10	Day 12
	AVG ± SD						
	(μg/g)						
Control	9.38 ± 0.09	7.46 ± 0.12	5.92 ± 0.21	4.26 ± 0.22	2.56 ± 0.07	0.92 ± 0.09	0.70 ± 0.03
LDPE/10HNT	-	7.86 ± 0.06	6.72 ± 0.12	4.92 ± 0.10	3.42 ± 0.12	1.50 ± 0.16	1.04 ± 0.03
LDPE/10TO@HNT	-	8.32 ± 0.03	7.50 ± 0.18	5.90 ± 0.24	4.72 ± 0.21	2.16 ± 0.16	1.80 ± 0.06
	ANOVA						
F	-	81.723	61.590	52.643	167.368	58.918	475.449
*p*	-	0.000	0.000	0.000	0.000	0.000	0.000

## Data Availability

The datasets generated for this study are available on request to the corresponding author.
